# Costs of patients with chronic kidney disease in Germany

**DOI:** 10.1371/journal.pone.0231375

**Published:** 2020-04-24

**Authors:** Afschin Gandjour, Wencke Armsen, Wolfgang Wehmeyer, Jan Multmeier, Ulrich Tschulena

**Affiliations:** 1 Frankfurt School of Finance & Management, Frankfurt, Germany; 2 Fresenius Medical Care Deutschland GmbH, Bad Homburg v.d.H., Germany; 3 Elsevier Health Analytics, Berlin, Germany; Murdoch University, AUSTRALIA

## Abstract

**Background:**

This study aimed to determine the costs and distribution of healthcare spending of patients with chronic kidney disease (CKD) at stages 3 and 4 and on dialysis both at the individual and population level in Germany.

**Methods:**

The study took the perspective of the German statutory health insurance (SHI) system and analyzed claims data on 3,687,015 insurees from the year 2014. To extrapolate costs to the whole SHI population, a literature search on the prevalence of CKD was conducted.

**Results:**

Average costs per person per year in an age- and gender-matched control group of the normal population were €2,876 (95% confidence interval [CI], €2,798 to €2,955) and ≥2.8-fold higher in CKD patients (€8,030 [95% CI, €7,848 to €8,212] at CKD stage 3, €9,760 [95% CI, €9,266 to €10,255] at CKD stage 4, and €44,374 [95% CI, €43,608 to €45,139] on dialysis). At CKD stages 3 and 4 the major cost driver was hospitalizations, contributing to more than 50% of total expenditures. Among dialysis patients, hospitalizations and dialysis-treatment costs contributed to 23% and 53% of total healthcare spending, respectively. At CKD stages 3 and 4, patients with the highest 20% of healthcare spending showed a considerable increase in per-patient costs over the reference population, while the bottom 80% of patients generated only moderately higher per-patient costs (p < 0.001). Comparing total CKD costs to total SHI expenditures yields that 10.2% of SHI expenditures was driven by patients at CKD stages 3 and 4 and 1.6% by dialysis patients.

**Conclusions:**

Healthcare spending of patients with CKD at stages 3 and 4 and on dialysis is concentrated among a small number of high-need patients. As hospitalizations and dialysis treatment are key drivers of total expenditures, strategies that lead to a reduction in hospitalizations, delay in dialysis onset, or increase in the availability of kidney donors should become important considerations by policymakers.

## Introduction

Chronic kidney disease (CKD) is defined as abnormalities of kidney structure or function, present for more than 3 months, with implications for health [1]. CKD is categorized into 5 stages depending on the level of the glomerular filtration rate (GFR) [1]. In the adult German population, CKD is estimated to have a prevalence between 2% and 7% [2,3]. The majority of CKD patients is classified as stage 3 [4]. A few international studies have compared the cost of end-stage renal disease (CKD stage 5) to that of earlier stages at an individual or population level [5,6,7,8,9]. These studies have consistently shown an increase in CKD cost with progression of disease. Still, evidence on the cost of earlier stages has been considered to be less reliable than evidence on the cost of end-stage renal disease, among others due to fewer diagnosed patients and less complete claims data [10]. To the best of our knowledge, a direct comparison of the cost of end-stage renal disease to that of earlier stages has not been conducted in Germany so far. In Germany, a few empirical studies exist with independent cost estimates either for end-stage renal disease or earlier stages [11,12,13]. The scope of evidence is particularly limited for earlier stages.

Based on a German claims data set, the aims of this study were (i) to determine the costs of patients with CKD at stages 3 and 4 and on dialysis both at the individual and population level, (ii) to identify the key cost drivers, and (iii) to describe the concentration of healthcare spending within each of these stages (the term ‘concentration of spending’ refers to the distribution of spending across the population). The analysis took the viewpoint of the German statutory health insurance (SHI), which covers approximately 87% of the German population or 72.2 million members [14]. In agreement with this perspective, the analysis only accounted for direct medical costs and excluded co-payments. For an overview on the German health care system, see the S1 File.

## Methods

### Population

**Fig 1:**
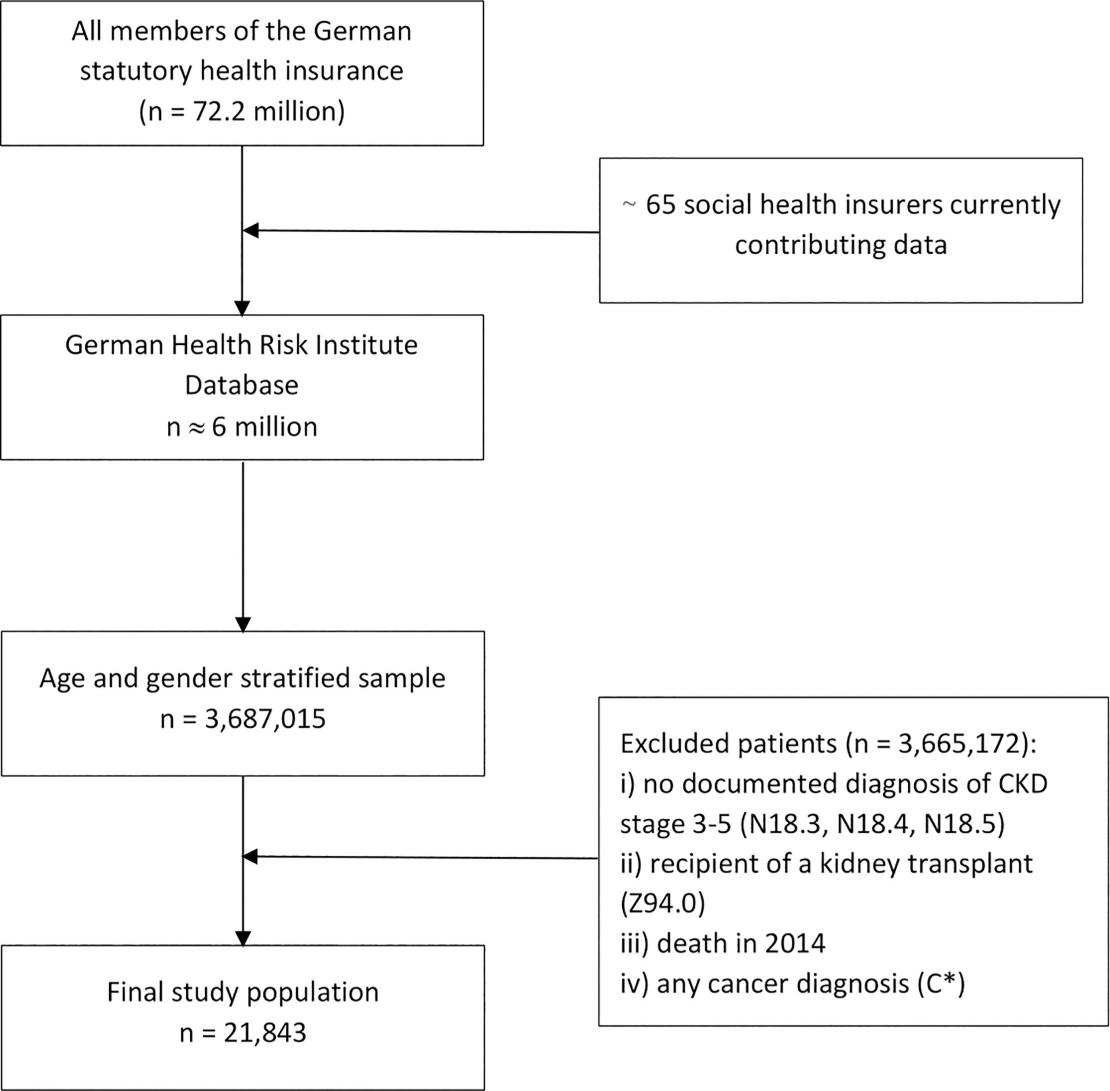
Flow diagram of patient selection.

A sample of 3,687,015 insurees who were enrolled in SHI funds was selected from the German Health Risk Institute (HRI) Database (see Fig 1 for a flow diagram of the patient selection process). The latter includes anonymized longitudinal claims data on healthcare services of approximately 6 million Germans covered by SHI. To this sample, we applied the following two inclusion criteria: (i) a documented diagnosis of CKD at stages 3 or 4 in ambulatory or inpatient care in adults (age > 18 years) in the first quarter of calendar year 2014, and ii) for documented CKD stage 5 start of dialysis at home (e.g., peritoneal dialysis) or in ambulatory (i.e., out of hospital), inpatient, or day care. Specifically, we analyzed for the presence of CKD using the codes of the International Classification of Diseases, 10th German modification (ICD-10-GM). The ICD-10-GM allows identifying the presence of CKD by the code N18.-. Specifically, patients at CKD stages 3 and 4 were identified by ICD codes N18.3 (GFR 30–59 ml/min/1.73 m^2^ body surface area) and N18.4 (GFR 15–29 ml/min/1.73 m^2^), respectively. Those undergoing dialysis treatment were identified by a combination of ICD codes N18.5 (GFR < 15 ml/min/1.73 m^2^) and Z49*/Z99.2 (actual dialysis treatment). Note that Z49* not only includes extracorporeal dialysis but also other types of dialysis including peritoneal dialysis. ICD code Z99.2 refers to dependence on renal dialysis.

Dialysis costs comprised weekly dialysis fees paid to dialysis providers. Dialysis fees excluded reimbursement fees for physicians and medication costs, both of which were reimbursed separately (except for heparin given on the day of hemodialysis, which was covered by the dialysis fee). The level of the fee was dependent on the age of the patient (above or below 18 years), the type of dialysis (hemodialysis vs. peritoneal dialysis), and the number of patients per dialysis center.

The exclusion criteria of this study were: (i) death, ii) recipient of a kidney transplant (ICD code Z94.0), and (iii) cancer diagnosis (ICD code C*) in 2014 (regardless of the presence of a cancer-free period). The reason for the exclusion of decedents and cancer patients were the high costs both of end-of-life and cancer care, resulting in an increase in the variance of costs of CKD patients. Note that based on our inclusion and exclusion criteria, patients with CKD stage 5 who underwent conservative treatment, were recipients of a kidney transplant, and/or were considered to be on the verge of dialysis treatment or kidney transplantation were thus also excluded due to unreliable coding in this transition phase.

A control group without patients with CKD at stages 3 and 4 and on dialysis was matched for age and gender. Specifically, it was matched to the mean age and gender proportion of each CKD stage, thus minimizing differences in mean values.

### Individual costs

Costs were calculated separately for each of 7 domains—inpatient care, outpatient care, medications, dialysis therapy, therapeutic and medical aids, sick benefits (paid sick leave), and dentists. As all cost categories were considered at the patient level, they included costs unrelated to CKD. To determine per-patient outpatient costs, outpatient fees remunerated in points (according to the German ambulatory physician fee schedule) were transformed to euros by multiplying the sum of points for each patient with the national point value. Administrative and transportation costs were not included in the claims data set and hence could not be considered. These costs comprised 7.1% of total SHI expenditures in 2014 [14]. For each patient total cost was calculated as the sum of the 7 domains.

In order to assess the distribution of spending, we determined patients with the highest 5%, 10%, and 20% of individual healthcare costs in the reference group (without CKD) as well as at each CKD stage. We used a two-tailed unpaired t-test to compare high spenders with average spenders at each CKD stage with regard to age, gender, and prevalence of type 1 and 2 diabetes mellitus. Diabetic patients were identified by ATC (Anatomical Therapeutic Chemical) and/or ICD codes. While ATC codes allow the detection of antidiabetic drug prescriptions, ICD codes also enable the identification of diabetic patients without pharmaceutical treatment. In addition, we compared high spenders with average spenders at each CKD stage in terms of individual costs, contribution of hospitalizations to total individual spending, and share of cardiovascular hospitalizations. Finally, we analyzed the contribution of top healthcare spenders to total healthcare spending, total hospital spending, and total number of hospitalizations at each disease stage.

We considered p values < 0.05 to be statistically significant.

### Population costs

As a final step of our analysis, we extrapolated per-patient costs determined in our sample to the whole SHI population, thus calculating total expenditure in the German SHI for patients at CKD stages 3 and 4 and on dialysis. To this end, we multiplied mean per-patient costs at each stage with the corresponding prevalence. To identify studies on the prevalence of CKD stages 3 to 5 in the German general population, we conducted a search in PubMed on January 3, 2019 using the following algorithm: *(prevalence[Title] OR population[Title]) AND (kidney[Title] OR renal[Title] OR CKD[Title]) AND (glomerular filtration rate OR eGFR OR GFR) AND Germany*. In addition, we searched for meta-analyses on national or international prevalence estimates. To this end, we conducted another search in PubMed on January 3, 2019 using the following algorithm: *(prevalence[Title] OR population[Title]) AND (kidney[Title] OR renal[Title] OR CKD[Title]) AND (glomerular filtration rate OR eGFR OR GFR) AND meta-analysis*.

## Results

### Individual costs

The final sample of the analysis included 21,843 patients with CKD at stages 3 and 4 and on dialysis, with an average age of 73.7 years and a 49% share of males (see Table 1 for details). A cancer diagnosis was found in 18% of patients in the control group and in 25% of patients at CKD stages 3 and 4 and on dialysis. These patients were excluded. In the control group the mean age was 72.4 years and the percentage of men was 54%. The prevalence of diabetic patients identified by ATC and/or ICD codes was more than twice as high at each CKD stage (54%, 57%, and 46% at CKD 3, 4, and on dialysis, respectively) compared to the reference population (23%).

**Table 1:** Demographic characteristics of the sample population.

	CKD stage 3	CKD stage 4	Dialysis	Reference group
Sample size	16,290	3,469	2,084	13,969
Mean age (years)	75.8	77.2	68.1	72.4
Standard deviation of age (years)	11.0	11.6	14.7	11.9
Percentage male	45.5	42.3	58.1	54.2
Percentage diabetes (ATC code)	38.4	39.2	28.4	14.7
Percentage diabetes (ICD code)	53.7	56.6	45.7	22.2

ATC = Anatomical Therapeutic Chemical, ICD = International Classification of Diseases

Whereas in the reference group the 5%, 10%, and 20% of patients who contributed most to total healthcare expenditures were significantly older (p < 0.001), among CKD patients a significant relationship with age was only detected at CKD stage 3 (for the top 5% and 20% spenders, p < 0.01). That is, in advanced CKD age lost its significance as a driver of individual costs. For male gender the relationship with annual per-patient expenditures was similar: Only at CKD stage 3 being male was significantly associated with belonging to a high-spending group (top 5%, 10%, and 20% spenders; p < 0.05). For diabetes mellitus the relationship with individual spending was found to be overall stronger. At all CKD stages but also in the reference group the top 5%, 10%, and 20% of healthcare spenders had a significantly higher share of diabetes (p < 0.01) except for the top 5% of healthcare spenders at CKD stage 4.

As shown in Table 2 and Fig 1 of the S1 File, for patients at CKD stages 3 and 4 the major cost driver was hospitalizations, contributing to more than 50% of individual expenditures. In dialysis patients, costs of hospitalizations represented 23% of individual spending but were still higher than for patients at CKD stages 3 and 4 on an absolute level. For dialysis patients the main contributor to individual costs was dialysis care itself (53%). Table 2 also shows that individual costs at CKD stage 3 were already 2.8 times higher than those in the reference population. For patients undergoing dialysis they were even 15-fold compared to the reference group.

**Table 2:** Per person annual cost by disease stage and cost category.

	Reference group (n = 13,969)		CKD stage 3 (n = 16,290)		CKD stage 4 (n = 3,469)		Dialysis (n = 2,084)	
	Mean	Std.	Mean	Std.	Mean	Std.	Mean	Std.
**Hospital**	1,034 €	3,634 €	4,310 €	9,699 €	5,441 €	11,161 €	10,029 €	17,681 €
**Medication**	664 €	1,859 €	1,870 €	5,040 €	2,380 €	8,071 €	6,338 €	5,435 €
**Outpatient care**	686 €	658 €	1,112 €	747 €	1,215 €	748 €	3,826 €	1,302 €
**Dialysis**	0 €	0 €	27 €	1,251 €	0 €	0 €	23,341 €	7,331 €
**Therapeutic aids**	93 €	451 €	178 €	631 €	195 €	668 €	181 €	574 €
**Medical aids**	116 €	615 €	273 €	1,188 €	299 €	905 €	357 €	1,198 €
**Sick benefits**	29 €	354 €	39 €	497 €	36 €	492 €	61 €	582 €
**Dentist**	255 €	705 €	220 €	635 €	195 €	556 €	241 €	675 €
**Total**	2,876 €	4,747 €	8,030 €	11,872 €	9,760 €	14,869 €	44,374 €	17,831 €

CKD = chronic kidney disease, Std = standard deviation

**Fig 2:**
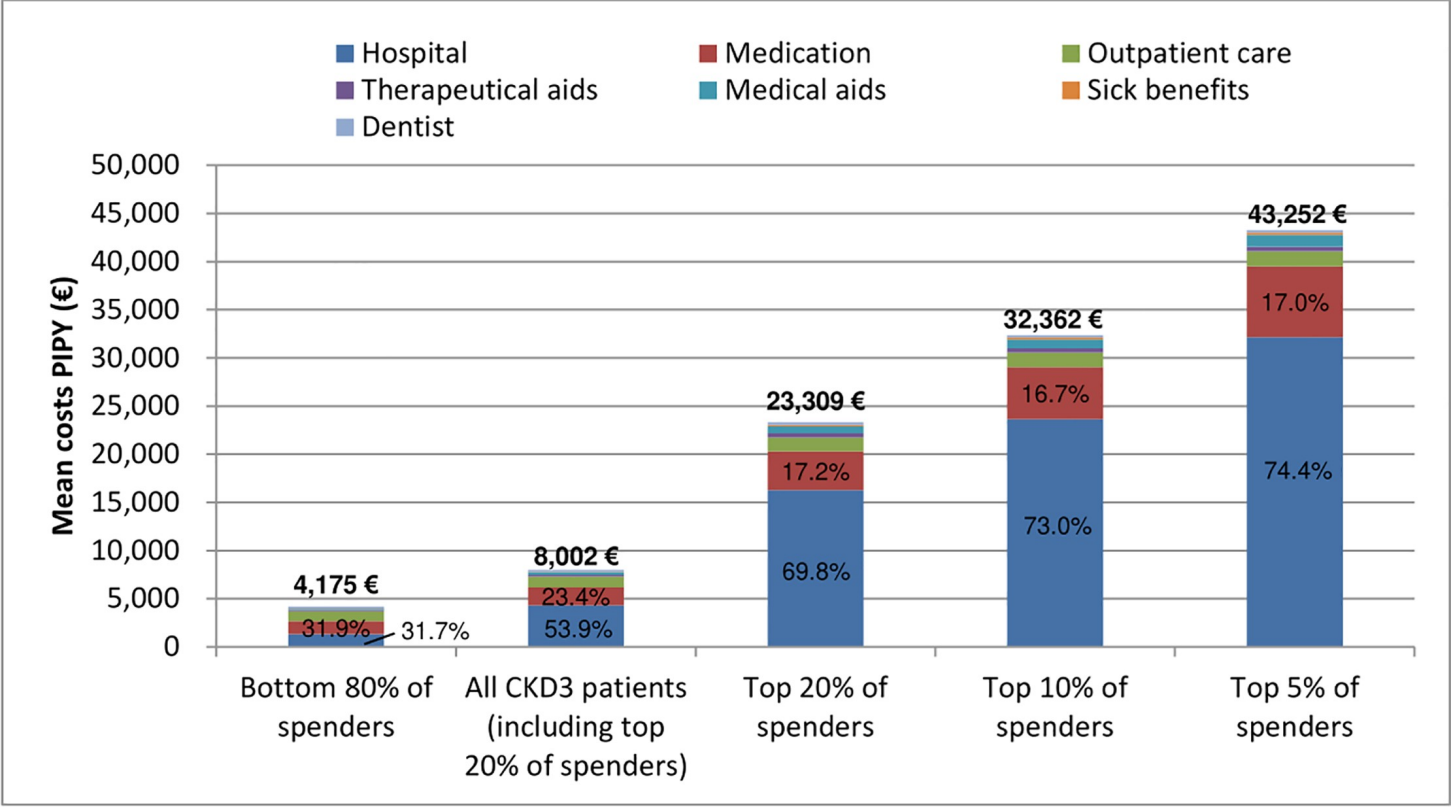
Concentration of healthcare spending at chronic kidney disease (CKD) stage 3. Percentages refer to share of hospitalization and medication costs by percentile of spending. PIPY = per individual per year, CKD = chronic kidney disease.

When specifically analyzing above-average spenders at CKD stage 3, we found that the top 5%, 10%, and 20% of healthcare spenders showed, respectively, a 5.4-, 4.0-, and 2.9-fold increase in individual costs compared to the average spender at stage 3 (Fig 2, p < 0.001). Noteworthy, the top 5% of healthcare spenders already incurred per-patient expenditures that were on par with those of the average spender on dialysis (€43,783 vs. €44,374, p = 0.30). Among high spenders at CKD stage 3, hospitalizations were a more important cost driver than for average spenders: Among the top 20% of healthcare spenders, hospitalizations contributed to almost 70% of the total individual cost, and among the top 5% of spenders they even accounted for 74% of the total. Notably, the bottom 80% of spenders incurred mean costs that were just 1.5 times higher than in the reference population.

**Fig 3:**
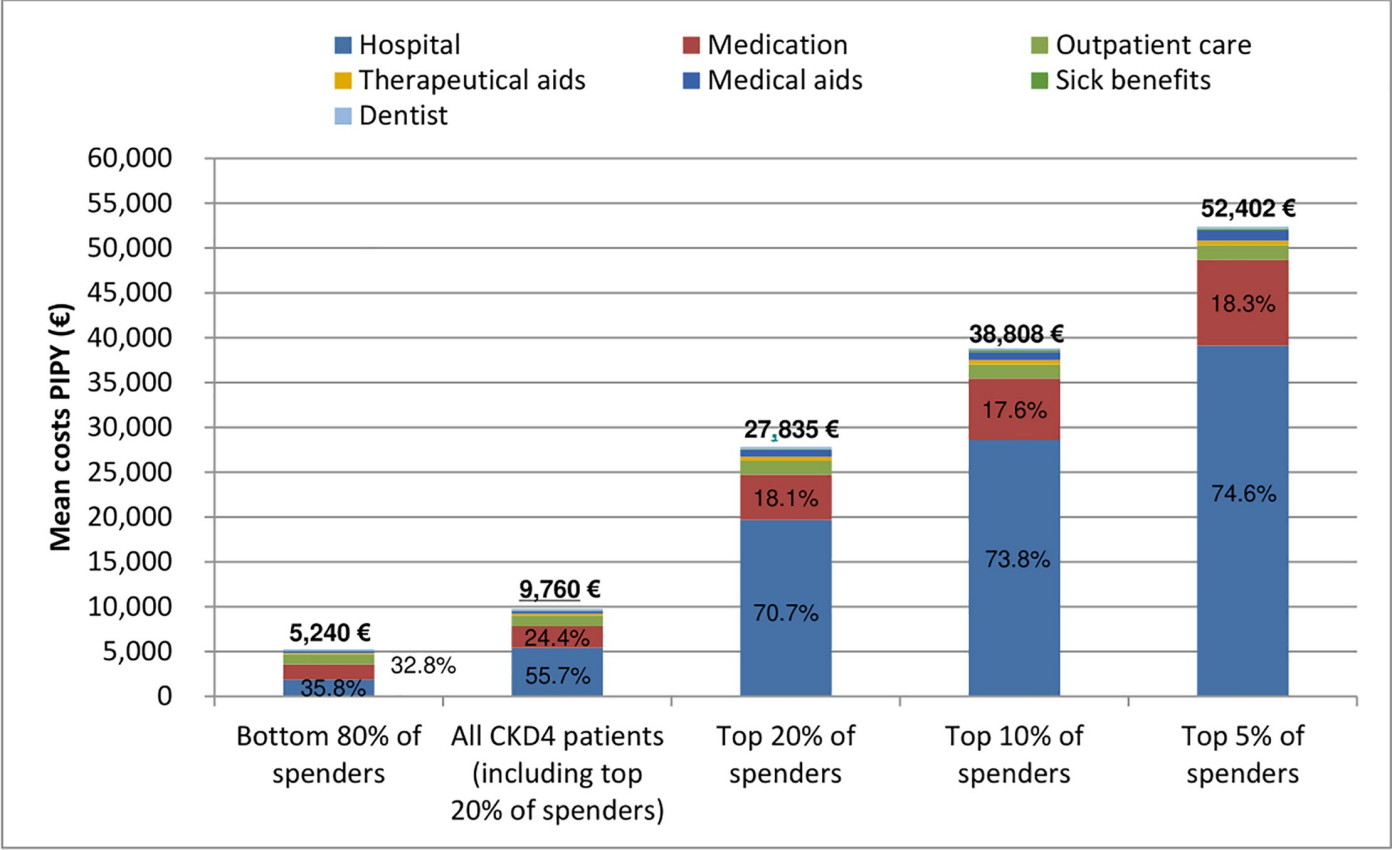
Concentration of healthcare spending at chronic kidney disease (CKD) stage 4. Percentages refer to share of hospitalization and medication costs by percentile of spending. PIPY = per individual per year, CKD = chronic kidney disease.

At CKD stage 4, the top 5%, 10%, and 20% of healthcare spenders showed, respectively, a 5.4-, 4.0-, and 2.9-fold increase in costs compared to the average spender at that stage (Fig 3). Noteworthy, the top 5% of healthcare spenders incurred higher annual per-patient expenditures than the average patient on dialysis treatment (€52,402 vs. €44,374, p < 0.01). Similar to CKD stage 3, among high spenders, hospitalizations were a more important cost driver than among average spenders: Among the top 20% of healthcare spenders hospitalizations contributed to more than 70% of the total per-patient cost, and among the top 5% of spenders they accounted for even three quarters of the total. The bottom 80% spenders incurred mean individual costs that were 1.8 times higher than in the reference population.

**Fig 4:**
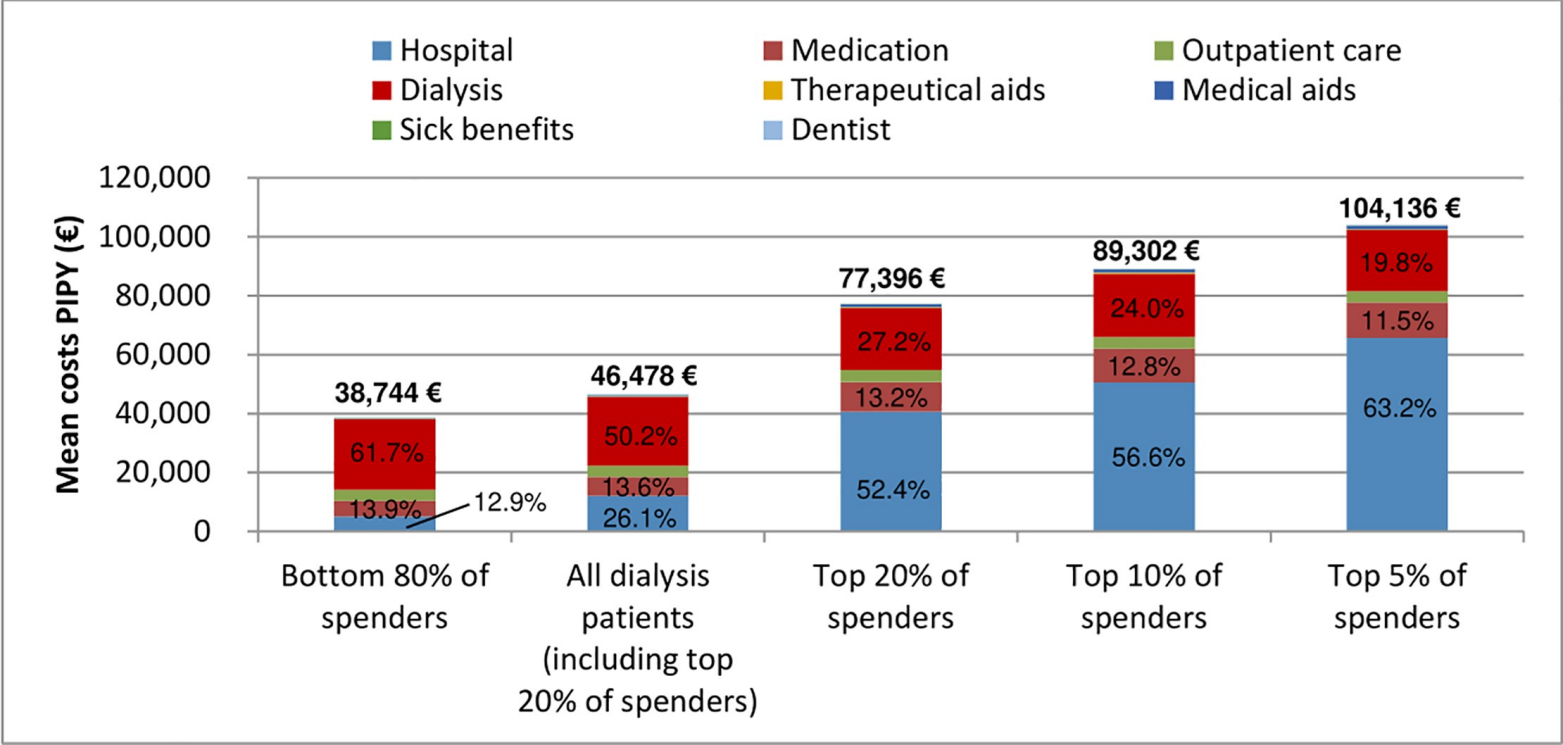
Concentration of healthcare spending among dialysis patients. Percentages refer to share of hospitalization, medication, and dialysis costs by percentile of spending. PIPY = per individual per year.

Among dialysis patients the top 5%, 10%, and 20% of healthcare spenders showed, respectively, a 2.2, 1.9-, and 1.7-fold increase in individual costs compared to the average spender (Fig 4, p < 0.001). The relative cost increase was therefore attenuated compared to earlier stages, owing to the high needs of the dialysis patient population and the high fixed costs of the dialysis procedure itself. This led to less concentration of spending in the dialysis population. Similar to earlier stages though, the contribution of hospitalizations to total individual spending was larger for high spenders than for the average spender. Among high spenders, hospitalizations led to even higher individual costs than the dialysis procedure itself (Fig 4). Still, the relative contribution of hospitalizations was smaller than for high spenders at CKD stages 3 and 4.

The contribution of top healthcare spenders can also be analyzed from an aggregated cost perspective (Fig 2 of the S1 File): At each disease stage, total healthcare spending was concentrated among a subset of insurees. For dialysis patients, spending was less concentrated than at CKD stages 3 and 4, however. The reason was a smaller difference in per-patient annual cost between high- and average-spenders (see Fig 4).

When analyzing concentration of spending specifically for different cost categories, total hospital spending was most concentrated at each CKD stage, i.e., more than total healthcare spending. For patients with CKD at stages 3 and 4 and on dialysis the top 20% of hospital cost spenders contributed to 75%, 72%, and 67% of total hospital spending, respectively (Fig 3 of the S1 File). In contrast, the least concentrated cost category was outpatient cost, with the top 20% of spenders contributing to just 26%, 26%, and 21% of total outpatient spending, respectively. The underlying reason were fixed outpatient fees regardless of the intensity of outpatient care. Medication costs were also less concentrated than total healthcare spending, with the top 20% of spenders contributing to 43%, 42%, and 32% of total medication spending, respectively.

### Hospitalizations

As shown in Fig 4 of the S1 File, the number of hospitalizations increased with CKD progression and percentile of spenders. Noteworthy, the top 20% of spenders at CKD stage 3 already incurred more hospitalizations than the average patient receiving dialysis care.

Still, the number of hospitalizations was less concentrated than hospital spending: For patients with CKD at stages 3 and 4 and on dialysis the top 20% of healthcare spenders contributed to 38%, 35%, and 31% of total hospitalizations, respectively. One reason for the larger concentration of hospital spending compared to concentration of number of hospitalizations was a trend towards longer length of stay of top healthcare spenders at each disease stage, leading to relatively high individual spending.

Patients with at least one cardiovascular hospitalization accounted for 43%, 44%, and 38% of patients with CKD at stages 3 and 4 and on dialysis, respectively. Among the top 20% of spenders at each stage the share of patients with at least one cardiovascular hospitalization increased further (57%, 66%, and 57% respectively; p < 0.001).

### Population costs

Finally, we extrapolated the mean cost estimates of our sample to the whole SHI population in 2014 (70.3 million insurees [14]), thus calculating total SHI expenditure of patients with CKD at stages 3 and 4 and on dialysis. Our literature search for German studies on the prevalence of CKD stages 3 to 5 yielded 44 hits, out of which we obtained three relevant studies [15,2,3]. We chose the study by Trocchi et al. [3] due to its more recent study period and better age matching with the general population in Germany in the year 2014 (the average age in that study was 47 years compared to 44 years in the general population [16]; the average age in the studies by Aumann et al. [2] and Zhang et al. [15] was 56 years and 62 years, respectively). In any case, prevalence estimates reported by Aumann et al. [2] (3.1% and 5.9%) fell in-between the bounds reported by Trocchi et al. [3], which were 2.1% and 6.9%. The bounds reported by the later study stemmed from different estimation equations for GFR (Chronic Kidney Disease Epidemiology Collaboration cystatin C equation and Full Age Spectrum creatinine equation, respectively). Given the lack of information on the utilization of the different types of estimation equations for GFR in the German SHI, we chose the arithmetic mean of the bounds reported by Trocchi et al. [3] (4.5%) as our base-case estimate and tested the bounds in a sensitivity analysis.

The German prevalence studies were limited by providing aggregate estimates for CKD stages 3 to 5, without breaking them down by stage (except for a stage 3 prevalence estimate reported by Zhang et al. [15]). Only the number of dialysis patients can be estimated fairly accurately in Germany. In the German SHI system, approximately 75,000 patients receive continuous ambulatory dialysis [17]. In order to obtain the number of patients at CKD stage 4, we applied a global prevalence estimate from the most recent review and meta-analysis of international studies [4] retrieved by our literature search strategy. As the global estimate for CKD stages 3–5 (8.1%) was higher than the mean of the bounds reported by Trocchi et al. [3] (4.5%), we calculated the relative share of patients at CKD stage 4 in the global study (4.9%) and applied it to the SHI population. To obtain the number of CKD stage 3 patients, we subtracted the estimates for patients at CKD stage 4 and on dialysis from the aggregated number of patients with CKD at stages 3 and 4 and on dialysis. The size of the SHI population was further adjusted downwards for the age group below the age of 18 [18], as the latter was excluded from the prevalence estimate by Trocchi et al. [3]. This age group comprised 16% of the German population in 2014. On the other hand, we did not exclude the population above the age of 79, although it was excluded by Trocchi et al. [3] as well, because the age gradient reported by Trocchi et al. [3] rather suggested an above-average prevalence in this population, implying an underestimate of the CKD population even upon inclusion. Prevalence data and SHI expenditure by stage are reported in Table 3. As shown, total expenditures on patients at CKD stages 3 and 4 are approximately 6 times larger than those on dialysis patients.

**Table 3:** Total statutory health insurance (SHI) expenditure by chronic kidney disease (CKD) stage.

	Mean per-patient cost, € (95% CI)	SHI prevalence (range)	Total cost, € (range)
**Stage 3**	8,030 (7848–8212)	2,446,295 (1,101,604–3,790,986)	19,644,066,150 (8,846,025,670–30,442,106,631)
**Stage 4**	9,760 (9266–10,255)	130,976 (61,122–200,830)	1,278,389,513 (596,581,773–1,960,197,253)
**Dialysis**	44,374 (43,608–45,139)	75,000	3,328,014,750 (3,270,596,072–3,385,433,428)

CI = confidence interval

## Discussion

Our SHI claims-data analysis shows that among patients at CKD 3 and 4 and on dialysis mean annual per-patient costs increase with disease progression and severity. Furthermore, at each disease stage healthcare spending is concentrated among a subset of insurees. In contrast, at CKD stages 3 and 4 the bottom 80% of patients generate only moderately higher per-patient costs than the reference population. At CKD stages 3 and 4, hospitalizations are the key cost driver and patients are frequently admitted based on cardiovascular diagnoses. While our study excluded deaths within the analysis period of 12 months, health expenditures typically start rising even before the last year of life [19]. Based on the association of CKD with higher mortality [20], this rise can at least partially explain the excess costs of CKD patients compared to the reference population. To what degree cost differences between the different stages are influenced by the exclusion of deaths in our study depends on the size of the excluded population at each stage (which increases with CKD severity [20]) and the costs of decedents compared to those of survivors at each stage. In any case, the excess costs of CKD patients is not explainable by patients whose kidney function has returned to normal after CKD documentation in the first quarter of 2014 because these patients are included in the annual per-patient cost of CKD and thus cause a downward bias.

Per-person spending in the reference group compares well with that of the average German SHI insuree. In 2014, non-administrative healthcare costs per SHI insuree were €2,755 [14], which is comparable with €2,876 in our reference group. Also, annual per-patient expenditures on hospitalizations show little difference (€965 vs. €1,034). These results support the generalizability of our findings to all SHI insurees.

A few German cost analyses on patients with CKD have been published in the academic literature. Similar to our study, Baumeister et al. [12] demonstrated an increase in per-patient healthcare expenditure at CKD stages 3 and 4 compared to absence of CKD (the relative increase fell in-between the relative increase observed for CKD 3 and 4 in our study). While their study relied on resource consumption patterns revealed from a patient survey, it included costs unrelated to CKD, similar to our study. In a cost analysis on dialysis patients, Icks et al. [13] used patient medical records to identify resource consumption patterns. While their study was also conducted from the perspective of the SHI, it purposely focused on dialysis-related costs only. On the other hand and in contrast to our study, Icks et al. [13] accounted for the cost of transportation and showed a contribution of 7.6% to total dialysis-related costs. Furthermore, while our study excludes deaths within the next 12 months and cancer patients and thus intentionally does not account for costs of end-of-life and cancer care, they were included in the analysis by Icks et al. [13]. Perhaps noteworthy, in our study annual per-patient costs of the dialysis procedure itself plus total outpatient physician expenditure are lower than in the study by Icks et al. [13] (€27,167 vs. €30,029). This holds despite the fact that our study even includes outpatient physician expenditure for dialysis-unrelated care. Most likely, this decline can be attributed to a reduction in fees for regular dialysis visits in 2013. In another, earlier dialysis-cost study from Germany, Kleophas and Reichel [11] estimated the cost per dialysis patient for the year 2002. Similar to our study, this study included all costs of dialysis patients including those unrelated to dialysis and excluded transportation costs. Yet, it did not rely on patient-specific data but used aggregated data and expert opinion. The dialysis procedure itself accounted for a larger share of total costs than in our study (62% vs. 53%).

Results of our analysis are also comparable to the international literature. For example, based on claims data from Italy, Roggeri et al. [9] reported costs of €38,821 for patients in their first year of dialysis, which are similar to €44,374 per patient year calculated in our study (but independent of the time since commencement of dialysis). Another Italian study [21] demonstrated an increase in direct medical costs of CKD due to the presence of diabetes mellitus or cardiovascular disease, similar to what is found in our study. In a prospective study in 18 countries, Kent et al. [22] showed that cardiovascular events were a major driver of CKD hospital costs, similar to our finding with regard to the share of hospitalizations by patients with a cardiovascular admission. Furthermore, a recent study on Chinese adults in Hong Kong [23] yielded cost multipliers of 1.89 and 4.16 for CKD stages 3 and 4, respectively, compared to a reference population without CKD and cardiovascular disease after adjustment for age and gender. In our study, the respective multiplier is higher for stage 3 (2.79) but lower for stage 4 (3.39), bearing in mind that our reference group still includes patients with cardiovascular disease (but to a lesser extent than in a population inclusive of CKD patients, which has a higher prevalence for cardiovascular disease).

In contrast to our study, a modelling study using secondary data from the English National Health Service [5] showed that costs of renal replacement therapy and CKD stage 3 to 5 are almost the same (whereas in our study costs of patients at CKD stages 3 and 4 in combination are 6 times larger than costs of patients on dialysis). One reason for the difference may lie in the fact that in contrast to our study, Kerr et al. [5] did not include all expenditures unrelated to CKD but only costs “for ‘excess’ events, above the level expected for a matched population without CKD.” This exclusion may have disproportionately affected CKD stages 3 and 4 due to their considerably higher prevalence. Furthermore, our study did not include the cost of transplantation and dialysis transportation, while in the study by Kerr et al. [5] they contributed to more than one third of the cost of renal replacement therapy. In this regard, it seems worth emphasizing that results of our study are really specific to the German SHI system and may not be transferable to other countries.

Based on prevalence estimates gathered from the literature, €20.9 billion representing 10.2% of total SHI expenditures are driven by patients with CKD stages 3 and 4, and 3.3 billion € representing 1.6% of total SHI expenditures are driven by dialysis patients. In total, this amounts to approximately 12% of total SHI expenditures in the year 2014, which were €206 billion in total [14]. While this analysis carries some uncertainty in particular due to the underlying prevalence estimate, two German prevalence studies [2,3] arrive at the same mean prevalence rate. It is again important to point out that the aggregate estimate does not represent the cost of CKD per se but the cost of CKD patients and as such captures costs of comorbidities such as diabetes or cardiovascular disease. On the other hand, as patients with a cancer diagnosis were explicitly excluded from our analysis, costs of CKD patients are even underestimated. Results of our population-cost analysis seem to be in agreement with an estimate of the share of CKD costs reported in the U.S. population above the age of 65 (Medicare), which is 25% even excluding the cost of end-stage renal disease [24].

In summary, CKD is an insidious disease that, from the perspective of an individual patient, remains rather an abstract disease entity until the inception of dialysis therapy [25]. Yet, as our study shows, from a payer’s perspective, the economic burden of CKD is identifiable at a much earlier stage. Given that more than 10% of SHI expenditures are associated with patients at CKD stages 3 and 4 and on dialysis, we suggest that German policymakers pay more attention to the needs of this population. Based on the uneven distribution of total healthcare expenditures and, in particular the concentration among a subset of insurees, programs that identify high spenders and coordinate their care seem a reasonable step forward. For patients with a frequent number of cardiovascular hospitalizations such programs may include the use of home monitoring. Given the contribution of co-morbidities such as diabetes mellitus and cardiovascular disease to CKD spending, CKD patients with these co-morbidities may especially benefit from enrollment in a chronic care program. Programs particularly aiming at early enrolment have a potential for cost savings by delaying the progression of CKD and avoiding renal replacement therapy [26]. In addition, primary prevention addressing lifestyle factors such as tobacco smoking, obesity, and lack of exercise could even reduce the incidence of CKD [27]. In this regard, it may be worth mentioning that spending on primary prevention and health promotion currently contributes to just 3% of total health care expenditures in Germany [28]. Increasing the availability of kidney donors may be a further measure to decrease the cost of end-stage kidney disease. In 2018, Germany had the lowest number of deceased donors (any organ) per million population among the eight Eurotransplant member states [29]. At the time of writing, German policymakers are debating ways to get more organs for the thousands of people on transplant waiting lists. Finally, policymakers may encourage a switch from in-hospital haemodialysis to less costly dialysis modalities such as at-home haemodialysis, peritoneal dialysis, and self-care dialysis, at least in more autonomous and healthier (or less comorbid) patients. This may require a change in the existing financial incentives for providers.

## Supporting information

S1 File Overview on the German health care system [30,31]. (DOCX)
