# Supplementary material for: Costs of patients with chronic kidney disease in Germany
Source: PLoS One. 2020 Apr 24;15(4):e0231375. doi: 10.1371/journal.pone.0231375 (PMC7182232; doi:10.1371/journal.pone.0231375)
Supplement: S1 File — (DOCX) [file pone.0231375.s001.docx]

**Supplementary File**

*Overview on the German health care system*

The following brief overview on the German health care system is excerpted from the following two sources: Blümel and Busse and Busse et al. [30,31]

The German Chancellor Otto von Bismarck introduced in 1883 the first social health insurance system in the world. This law defined the founding principles of today's statutory health insurance (SHI). According to the principle of solidarity, the size of the insurance contributions is based on the ability to pay; in turn, the insured individual is entitled to benefits according to need. Second, SHI is compulsory insurance in which employers take part in the financing. And finally, SHI is based on self-governing structures, which means that competencies are delegated to membership-based, self-regulated organizations of sickness funds and health-care providers.

All employed citizens and other groups, such as pensioners and individuals earning less than the opt-out threshold (€57,600 per year in 2017), have mandatory SHI, and their non-earning dependents are insured free of charge. Individuals with a gross income that exceeds the threshold and people who are self-employed can keep SHI on a voluntary basis or purchase substitutive private health insurance. About 87% of the population receive their primary coverage through SHI.

General practitioners (GPs) and specialists in ambulatory care who get reimbursed by SHI are by law mandatory members of regional associations that negotiate contracts with sickness funds. Regional associations of SHI-accredited physicians are responsible for coordinating care requirements within their region and act as financial intermediaries between the sickness funds and the physicians in ambulatory care. However, ambulatory physicians typically work in their own private practices.

Fig 1. Mean per person annual cost by disease stage and cost category. Percentages refer to share of hospitalization, medication, and dialysis costs by disease stage.

**2,876 €**

**8,030 €**

**9,760 €**

**44,374 €**

52.402€

PIPY = per individual per year

CKD = chronic kidney disease

Fig 2. Contribution of top healthcare spenders to aggregated total healthcare spending by disease stage.


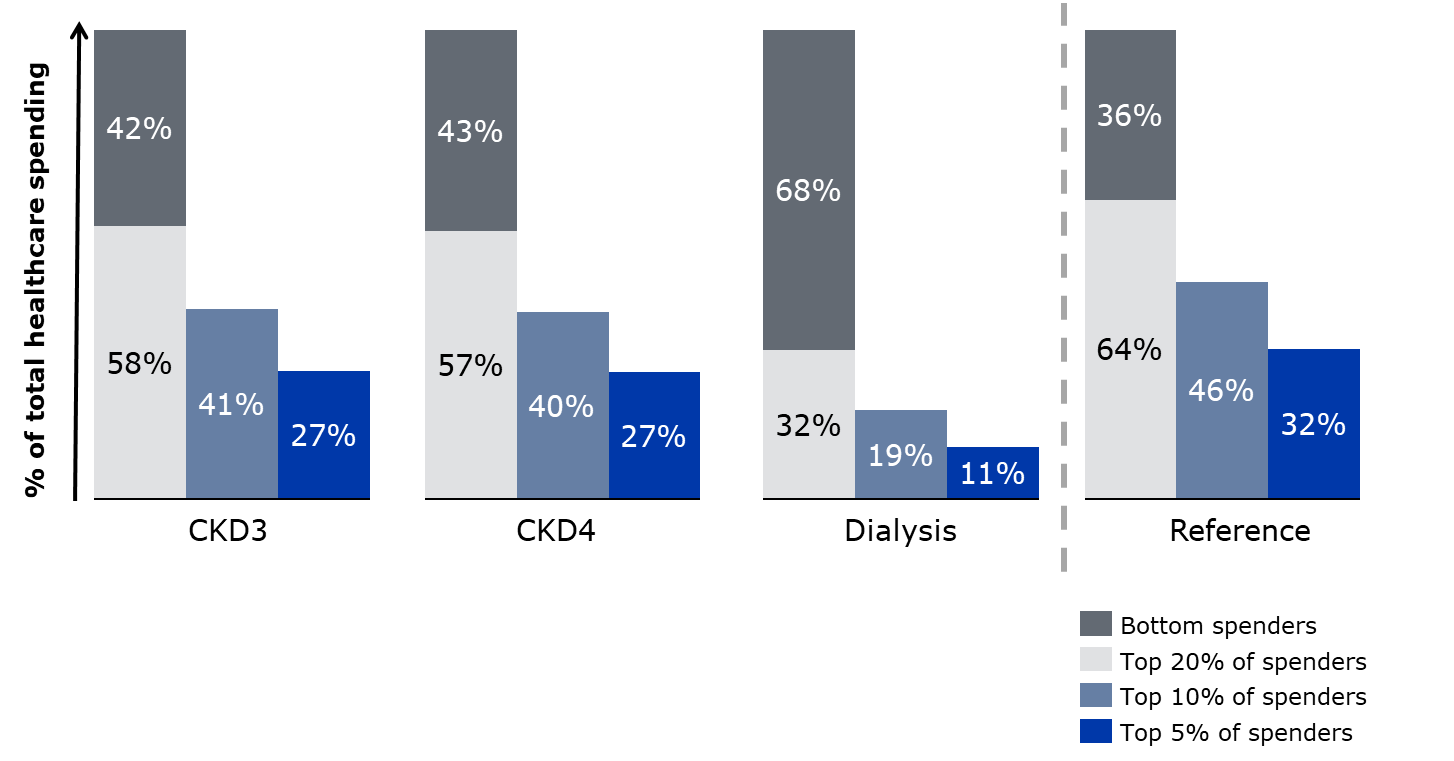


CKD = chronic kidney disease

Fig 3. Contribution of top healthcare spenders to aggregated hospitalization costs by disease stage.


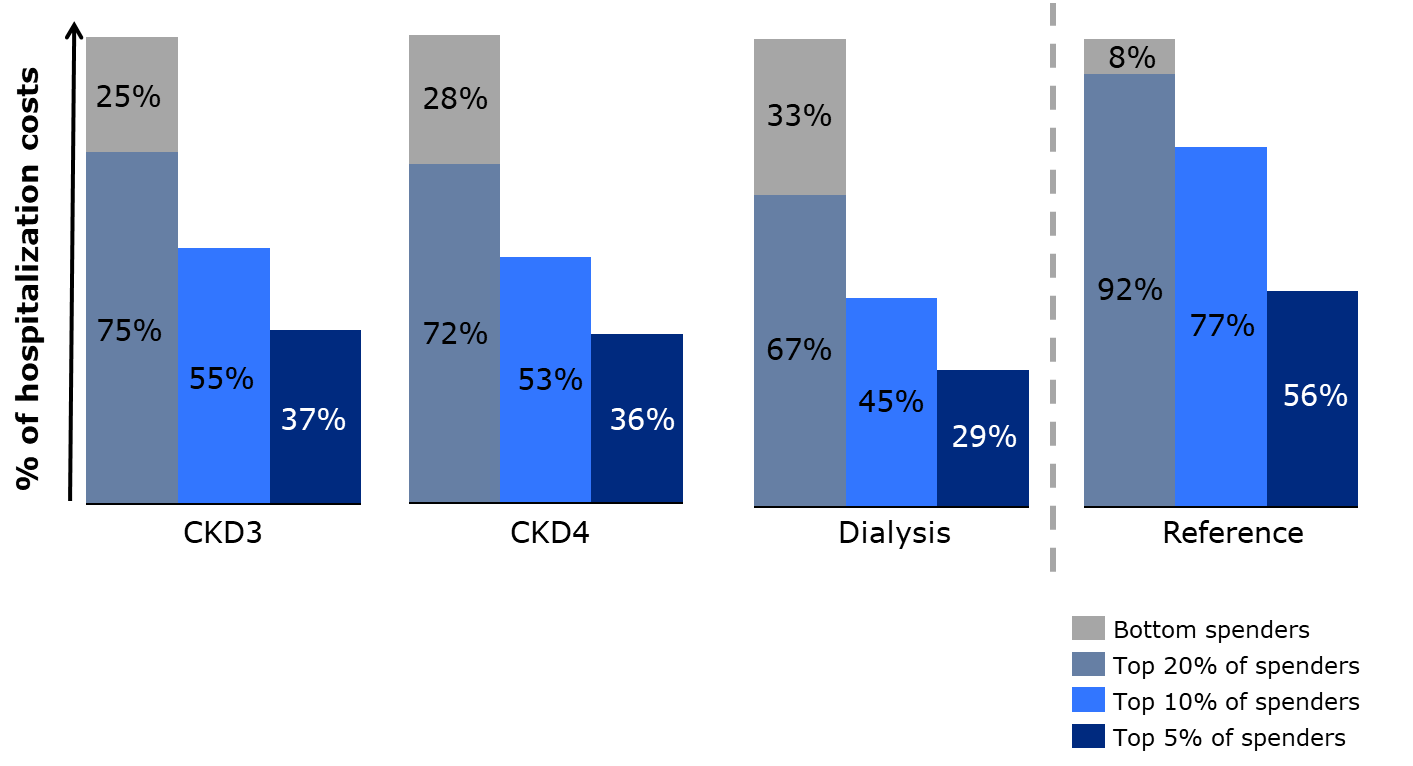


CKD = chronic kidney disease

Fig 4. Number of hospitalizations by disease stage.

PIPY = per individual per year

CKD = chronic kidney disease
